# Exploring the use of nutrition professionals in the WIC workforce: a landscape policy analysis

**DOI:** 10.3389/fpubh.2026.1941155

**Published:** 2026-09-21

**Authors:** Esther Annan, Aqsa Iqbal, Justin Y. Han, Elizabeth L. Fox, Genevive R. Meredith

**Affiliations:** 1 Cornell University Department of Public & Ecosystem Health, Schurman Hall, Ithaca, NY, United States; 2 Cornell University Public Health Program, Schurman Hall, Ithaca, NY, United States; 3 Cornell University Health Impacts Core, Atkinson Hall, Ithaca, NY, United States

**Keywords:** nutrition, paraprofessional, paraprofessional competence, WIC, workforce development and training

## Abstract

**Introduction:**

Workforce shortages can challenge the delivery of public health service. To ensure access to high-impact services in the Special Supplemental Nutrition Program for Women, Infants, and Children (WIC), there is interest in exploring expanded staffing models to support the delivery of nutrition services. We aimed to explore if or how paraprofessionals—staff without a nutrition/medical credential—are supporting the provision of WIC nutrition services in the U.S.

**Methods:**

We conducted a landscape analysis of publicly available WIC Policy and Procedure Manuals (PPMs) across a stratified sample of 23 WIC state agencies. Job titles, qualification requirements, delegated responsibilities, and workforce development pathways were extracted and analyzed.

**Results:**

A total of 242 unique job titles were identified and classified into 14 standardized workforce categories. Considerable variation was observed across jurisdictions in job titles, qualification requirements, delegated responsibilities, and competency-based training pathways. The term “paraprofessional” appeared in only seven of 23 state agency PPMs (30.4%) and in one standardized job title. As of January 2026, more than one-third (37.4%) of the unique nutrition-related postings (*n* = 107) did not explicitly require a college degree in the reviewed policy documents, indicating potential opportunities for paraprofessional staffing. These roles appear to support participant enroll-ment, nutrition services, benefit issuance, referrals, and administrative functions. Several state agencies also present structured training pathways that enable staff to assume progressive nutrition-service responsibilities.

**Discussion:**

Our work found that, in addition to WIC breastfeeding peer counselors, paraprofessionals are engaged in nutrition-service roles in at least some WIC state agencies, although roles and qualifications vary substantially. Further research is needed to identify promising practices and to inform workforce-development strategies that strengthen workforce capacity while maintaining quality of care.

## Introduction

The United States (U.S.) population experiences disparate health outcomes caused by variations in social and structural drivers of health (1–3). Pregnant and postpartum women, infants, and children are highly impacted: 28% of pregnant women face unmet social needs (1), resulting in health risks related to malnutrition, diabetes, and pregnancy complications (4–6). Further, some 17% of U.S. households are food insecure (7).

The U.S. Special Supplemental Nutrition Program for Women, Infants, and Children (WIC) is designed to reduce these risks by providing nutrition support to low-income pregnant, postpartum, and breastfeeding women, infants, and children (8, 9). For those eligible (8, 9), WIC provides supplemental foods, nutrition education, breastfeeding support, and referrals to other services (9). Current research shows that WIC helps reduce risks of adverse birth outcomes and infant mortality, reduce food insecurity, and support cognitive development and academic performance among children (10–12).

Despite these needs and benefits, WIC participation remains below eligibility estimates (10, 13–17). For example, in 2023, only 56.1% of an estimated 11.8 million eligible individuals participated in WIC (18); this suggests that millions of eligible women, infants, and children may not be realizing WIC’s nutritional and health benefits (16). Workforce models may be shaped by factors like capacity, organizational and professional actors. (Giusti and Persiani) (16, 17) WIC coverage gaps have previously been attributed to structural and programmatic barriers, including administrative complexity and hurdles in navigating eligibility requirements (16, 17). Research suggests that these challenges may be exacerbated by limited WIC staff numbers and capacity, and high staff turnover (16). Interventions to address these workforce gaps requires a broader understanding of the composition of the WIC workforce.

### WIC workforce

The U.S. Food and Nutrition Administration (FNA) oversees WIC, with administration delegated to 88 state agencies (including 50 state health departments, the District of Columbia, 32 Indian tribal organizations, and five territories) which oversee local agencies that deliver services through clinic sites (9). Under this structure, FNA sets regulations and policy; state agencies design programming to meet population needs, enabling adaptability, innovation, but also variation (19–21). For example, FNA sets minimum training requirements for staff roles and conducts compliance monitoring, and state agencies document staffing approaches, submitting reports to FNA (22, 23).

,Across local agencies, the WIC workforce is composed of credentialed staff [e.g., those holding a nutrition degree or a credential such as a registered dietitian (RD)], staff with a college degree, and those without a college degree (22). Current guidelines suggest that WIC local agencies must have credentialed staff to serve high-risk clients, including at least one designated Competent Professional Authority (CPA) to conduct assessments, determine nutritional risk, prescribe food packages, provide nutrition education, and make referrals (24). However, WIC sites report challenges recruiting and retaining credentialed nutrition professionals to fill some roles and meet client demand, especially in rural areas (25, 26). To address this need, there is interest in exploring broader engagement of paraprofessionals—staff without a nutrition/clinical credential—in the delivery of nutrition services to low-risk clients (27–31). While workforce shortages have generated interest in task sharing and paraprofessional models, less is known about how such models are reflected in formal workforce policies and how qualifications, functions, and competency pathways vary across jurisdictions. We set out to explore this.

## Methods

### Study design

To explore *if or how paraprofessionals are supporting the provision of WIC nutrition services in the U.S.* we used a policy landscape analysis. Specifically, we explored staffing models being used across WIC state agencies to support nutrition-service functions, and what roles or functions may be filled by paraprofessional staff. Focal questions included: what job titles are used; what qualifications are needed for each nutrition-related job title; and what nutrition-related functions can be implemented by a paraprofessional. As WIC workforce policies are administered by WIC state agencies, state agency Policy and Procedure Manual (PPM)s were selected as the primary data source.

### Sampling method

We developed a stratified sample of PPMs for our review, with strata focused on representation of geography (FNA regions), population size, and poverty level. Three state agencies were selected from each of the seven FNA regions (Mid-Atlantic, Mid-Western, Mountain Plains, Northeast, Western, Southeast, Southwest). We oversampled in two regions (Southeast and Southwest) (32) as they appeared to have comparatively larger number of state WIC agencies. We included one tribal nation and one district, and state agencies serving large, medium, and small populations (33), and populations with differing poverty levels (34).

### Data source identification

Between December 2024 and April 2025, a structured web search was conducted to identify publicly available WIC state agency PPMs within the sample frame. Searches were conducted using a combination of the State Agency name, WIC, and the words Policy and Procedure Manual, Operating Manual, Staffing Policy, State Plan. Where indicated, supplemental information was also reviewed from official agency websites.

### Data extraction

We reviewed each state agency PPM and extracted data into a multi-sheet Excel workbook. First, we reviewed the PPM for unique job titles, and the terms “paraprofessional” and logged findings in an Excel worksheet using titles and standard response categories (Y/N). We then extracted qualitative data verbatim for each job title, including required and recommended qualifications (e.g., education level, degree, professional license, and/or other trainings) and the function/responsibilities. Data extraction was conducted by three members of the six-person research team using a standardized extraction approach developed collaboratively by the team. To ensure completeness of data extraction, we also searched each PPM for the words “CPA,” “Certifier,” and “Competent Professional Authority.” If staffing roles related to these functions were listed in a different section of the PPM, we also extracted these data. Where paraprofessional training models were referenced, we reviewed the PPM and extracted details related to training pathways into an Excel worksheet.

To explore potential nutrition-related and paraprofessional-accessible positions, where no college degree is explicitly required, we examined the nutrition-related job titles that did not require a college degree, including those requiring high school diploma/GED or not listed. For each of these job titles, we then examined the job description and extracted key functions. Finally, to explore paraprofessional training models used across state agencies, we reviewed the qualitative data extracted and summarized the key themes found.

## Data analysis

We used content analysis to identify themes and cross-state variation for each research question. For each job title, we used a three-step process. First, we categorized each job title into one of three groups: nutrition services, breastfeeding services, or administrative services. For each job title initially categorized to “administrative,” we then reviewed the job description for details: if the position provided breastfeeding services or nutrition services, they were classified as such. Next, we standardized job titles based on similarity (e.g., categorizing “nutritionist” and “program nutritionist” and “qualified nutritionist” and “RD/RDN” into a standardized title of “Nutritionist”) to develop a list of unique, standard job titles. To ensure a standard approach, title standardization was completed by one researcher who discussed and demonstrated their approach to others on the research team for input and then alignment. Where the original/extracted title was ambiguous, the researcher looked at the full position description to inform interpretation of the title. Finally, job titles were appropriately categorized based on training needs, including: high school diploma, GED, associate/bachelor/master’s degree, dietitian/nutrition-related credential (e.g., RD, RDN), medical-related credential (e.g., MD, RN, PA, LPN), or not listed. Researchers worked together to establish consistent procedures for identifying relevant information. When information was unclear or not identified during initial extraction, a second team member reviewed the posting. Subsequently, 21 standardized categories were used upon an agreed classification approach, with ambiguous cases discussed and resolved by consensus (Figure 1). Descriptive analysis was done for themes in training qualifications required for each job title. Of note, titles related exclusively to breastfeeding services were excluded at this step from further analysis given FNA-direction to focus specifically on nutrition paraprofessionals.

**Figure 1:**
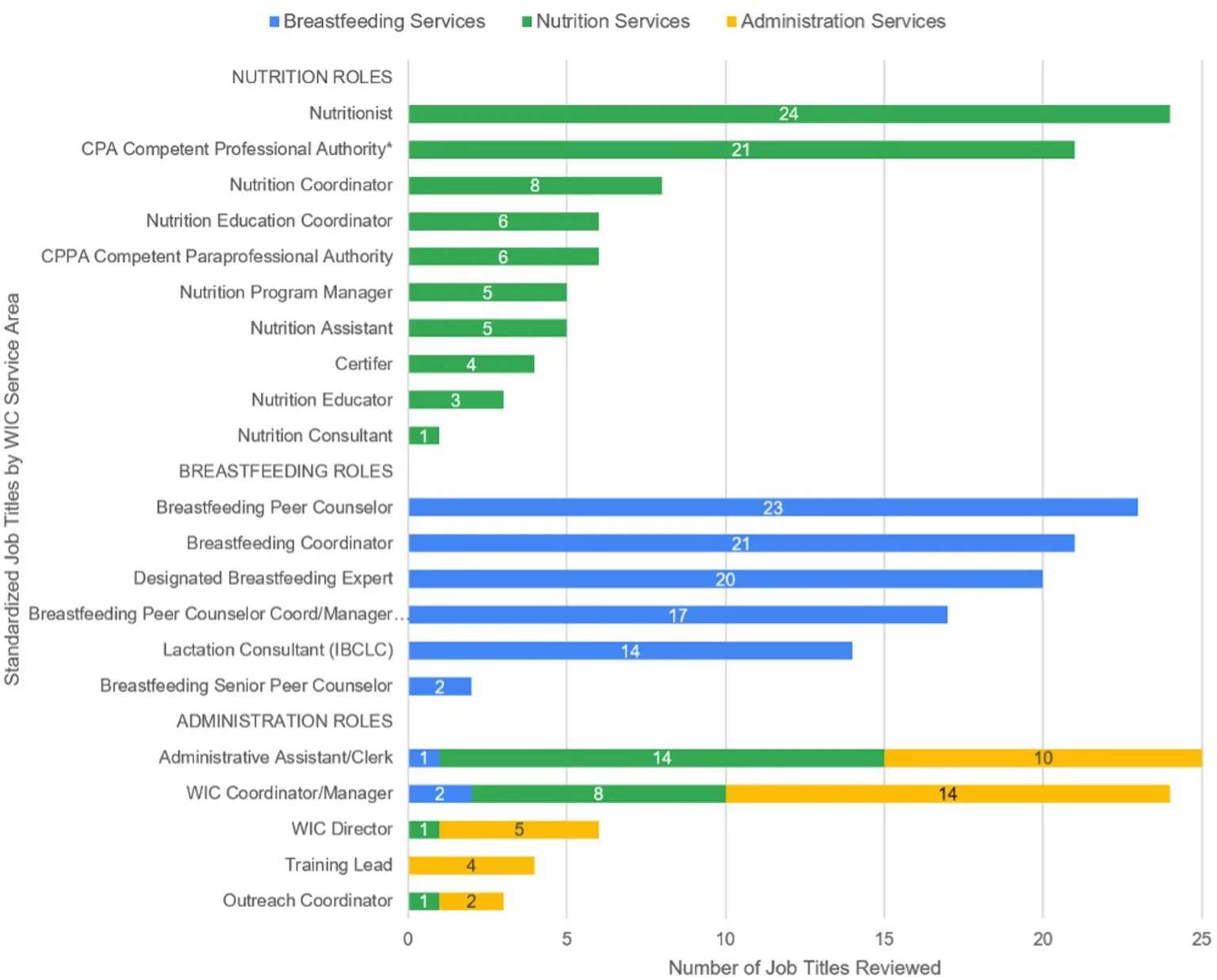
Distribution of standardized job titles by WIC service area.

## Results

### State agency characteristics

Our final data set comprised details from 23 of 88 WIC state agency PPMs (26.1%), representative of all seven USDA FNA regions, including some of the most populous U.S. states (California and Texas), some of the least populous states (Alaska, Washington DC), and some with the lowest (Minnesota, 5.6%; Utah, 5.6%) and the highest (New Mexico, 13.7%; West Virgina, 12.0%) proration of families living below the federal poverty line (Table 1).

**Table 1:** WIC state agency count, by nutrition, breastfeeding and administration roles.

**Region**	**WIC Agency**	**Population** ^**a**^	**Families Below Poverty (%)** ^**b**^	**Count of WIC Roles (#)** ^**c**^			
				**Nutrition**	**Breastfeeding**	**Administration**	**Total**
Mid-Atlantic Region	District of Columbia (DC)	689,545	11.90%	6	3	1	**10**
	Maryland (MD)	6,177,224	6.40%	2	6	0	**8**
	West Virginia (WV)	1,793,716	12.00%	6	1	0	**7**
Mid-West Region	Illinois (IL)	12,812,508	8.20%	5	4	0	**9**
	Michigan (MI)	10,077,331	8.69%	6	5	0	**11**
	Minnesota (MN)	5,706,494	5.60%	3	4	0	**7**
Mountain Plains Region	Colorado (CO)	5,773,714	5.90%	3	8	4	**15**
	Kansas (KS)	2,937,880	7.50%	3	4	0	**7**
	Montana (MT)	1,084,225	6.70%	4	3	0	**7**
Northeast Region	Massachusetts (MA)	7,029,917	6.70%	6	3	4	**13**
	Maine (ME)	1,362,359	6.40%	1	2	0	**3**
	New York (NY)	20,201,249	10.00%	5	4	0	**9**
Southeast Region	Alabama (AL)	5,024,279	11.30%	3	4	2	**9**
	Georgia (GA)	10,711,908	9.90%	6	8	2	**16**
	Kentucky (KY)	4,505,836	11.90%	5	5	1	**11**
	North Carolina (NC)	10,439,388	9.20%	1	4	1	**6**
Southwest Region	Inter-Tribal Council Arizona	296,000	n/a	6	3	3	**12**
	New Mexico (NM)	2,117,522	13.70%	5	1	1	**7**
	Texas (TX)	29,145,505	10.50%	5	5	1	**11**
	Utah (UT)	3,271,616	5.60%	6	6	2	**14**
Western Region	Alaska (AK)	733,391	6.50%	6	5	2	**13**
	California (CA)	39,538,223	8.50%	9	6	3	**18**
	Idaho (ID)	1,839,106	7.10%	5	6	8	**19**

a. U.S. Census (2020 population): https://data.census.gov/table/DECENNIALCD1182020.P1

b. NIH HDPulse: Social & Economic Environment. Poverty (Familes below poverty, 2020-2024): https://hdpulse.nimhd.nih.gov/data-portal/social/

c. Counts from State WIC Agency WIC Policy & Procedure Manual (web search).

### Nutrition-service workforce titles

Across the 23 State agencies reviewed, we extracted 242 unique job titles. The range of titles included in each state agency PPM was broad, from 3 in one state to 19 in another. Among the 242 titles, 44.2% (*n* = 107) appeared to be related to nutrition services, 41.3% (*n* = 100) to breastfeeding services and support, and 14.5% (*n* = 35) to administrative roles (Table 1).

After standardizing titles, we identified 21 unique job titles, including 10 that appeared to be exclusive to nutrition roles, 6 exclusive to breastfeeding roles, and 1 exclusive to administrative roles; 4 job titles appeared to be used across nutrition and/or breastfeeding and/or administrative roles showing variance across jurisdictions (Figure 1). Given the study’s focus on nutrition-specific paraprofessional roles, titles related exclusively to breastfeeding services and administration were excluded from further analysis.

### Qualification requirements

Among the 107 unique job titles/position descriptions related to nutrition services, our review showed that required qualifications varied substantially across jurisdictions. Specifically, 62.6% (*n* = 67) of the positions listed a requirement of some level of college degree (associates or higher) or a credential associated with university training (e.g., RD, nursing credential, other medical licensure; Figure 2); the remaining 37.4% (*n* = 40) did not specify requirement of a college degree, suggesting they could be filled by a paraprofessional.

**Figure 2:**
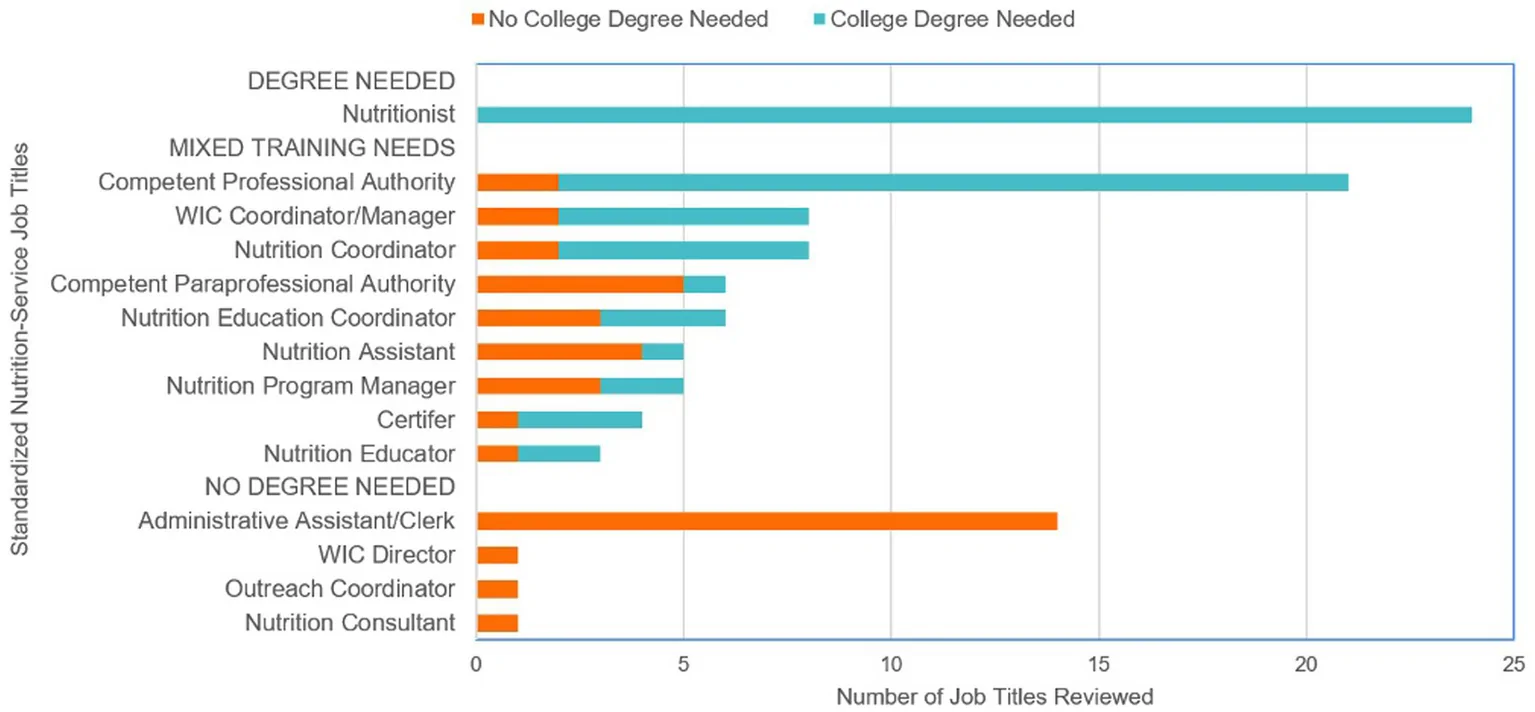
Education requirements per standardized nutrition-service job title.

Across all 23 jurisdictions, the “Nutritionist” role appeared to require a credential or a college degree; CPA roles appeared to require similar qualifications in 22 of 23 jurisdictions. The outstanding jurisdiction listed an alternative CPA certification pathway for those with a high school diploma, including competency-based training, mentoring, and evaluation).

Substantial variations existed in the qualifications required for other nutrition-service roles. For example, positions like Competent Paraprofessional Authority (CPPA), Nutrition Education Coordinator, Nutrition Assistant, and Nutrition Program Manager appeared to be open to individuals without a college degree in some but not all jurisdictions. Specifically, 10 of 23 jurisdictions reviewed (43.5%) listed nutrition-serving positions that seemed open to staff with a high school diploma or a GED, while four (17.4%) appeared to require at least a bachelor’s degree for all nutrition-related positions.

### Nutrition- service functions performed by non-degree staff

A total of 40 unique nutrition-related position descriptions met the criteria for review as “roles that may provide nutrition related services without requiring a college degree”. These positions were identified in 19 of 23 (82.6%) jurisdictions and included 13 standardized job titles (Figure 3). Thematic analysis of job descriptions identified 32 key functions implemented by these roles; we grouped these into three core service areas: client enrollment, client support services, and program administration (Figure 3).

**Figure 3:**
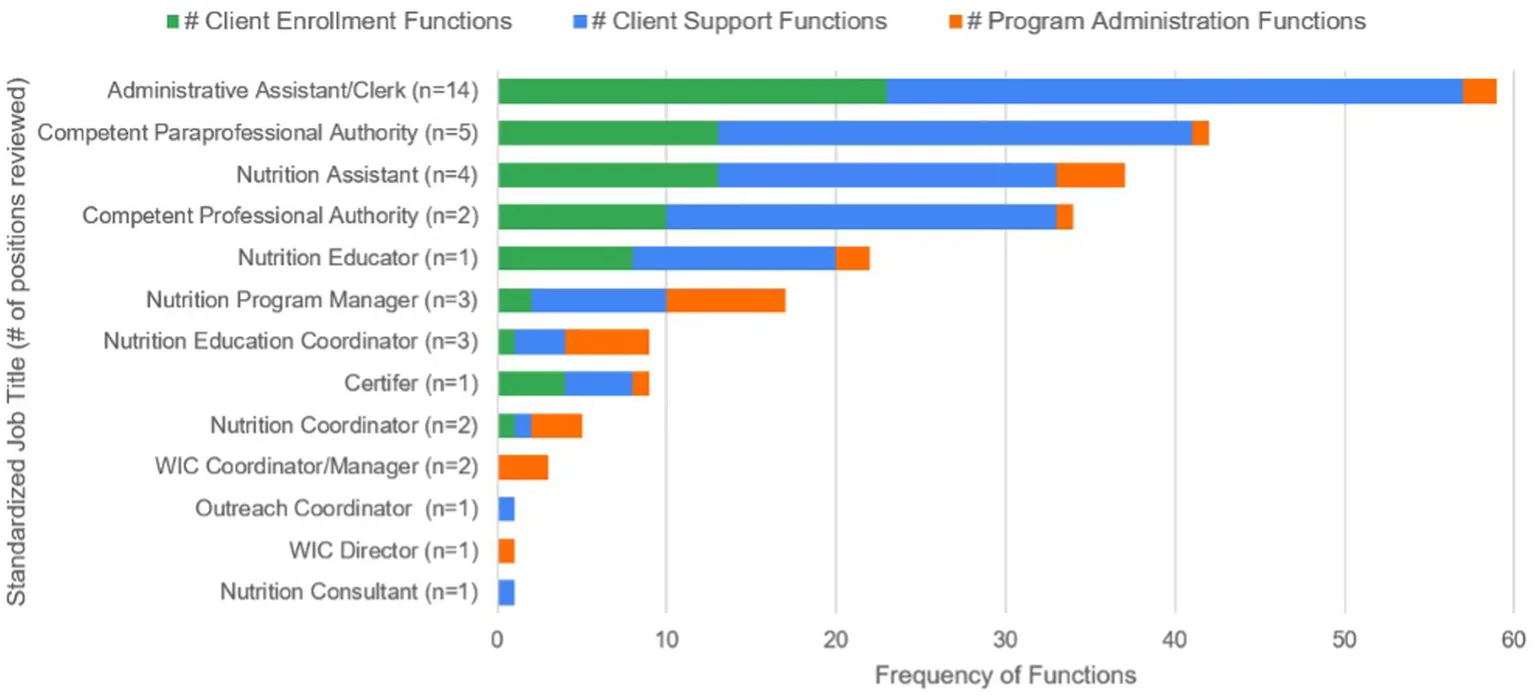
Frequency of job functions among standardized job titles, by function type.

Looking specifically at Client Enrollment functions, the most frequently noted tasks included screening for WIC eligibility, providing orientation to WIC and the benefits, completing anthropometric and hematological measurements, assessing nutrition risk, and conducting WIC certification (Figure 4). Within client support services, the most frequently noted tasks included making referrals, issuing food benefits, providing nutrition education, documenting client notes, scheduling and managing appointments, and assisting with reception. Program Administration functions centered on developing education materials, completing program evaluations, and promoting the WIC program in the community and to possible collaborators.

**Figure 4:**
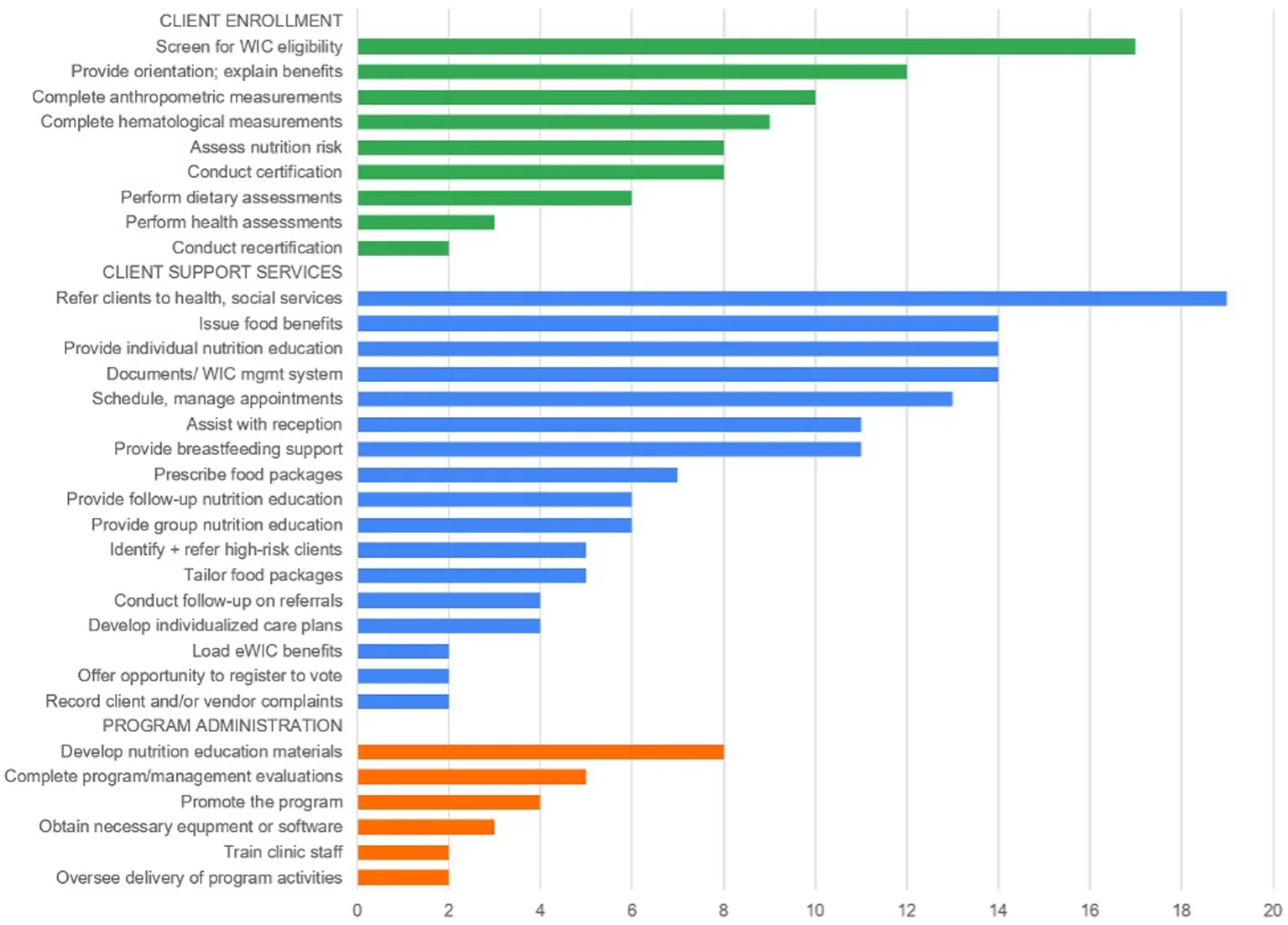
Frequency of role function among nutrition job titles.

### Competency-based training pathways

Six of 23 state agency PPMs (26.1%) listed training plans to upskill workers without a college degree or credential to take on CPA, CPPA, or similar roles. Across these jurisdictions, competency-based training plans appeared to focus on training paired with assessments and mentored/proctored practice; several described formal evaluative processes requiring paraprofessional staff to demonstrate competency before independently performing designated nutrition-service tasks. Examples of models include completing trainings and a series of competency checklists to qualify as a CPPA, allowing them to assess nutrition risk and prescribe supplemental foods under supervision; completion of a four-part curriculum (anthropometrics, hematological assessments, food package prescription, risk assignment, cultural humility, and identification of high-risk participants) to perform nutrition responsibilities in WIC clinics; a tiered model for CPA Assistants with trainings tied to specific tasks such as mid-certification visits and low-risk certifications; and completing training and demonstrating competence to become a Certifier with ability to determine nutritional risk and prescribe food packages.

## Discussion

WIC supports maternal and child health in the U.S (10–12), though its potential is not fully realized, in part due to gaps in the WIC workforce (8, 16, 17). Workforce challenges—including difficulties recruiting qualified staff and limited career advancement opportunities—have been well documented (16, 35). Other fields, such as education and healthcare have addressed similar workforce needs through paraprofessional models that expand workforce capacity and improve service reach (36–41). Aligned with this, and building on the success of WIC’s breastfeeding peer counselor paraprofessional model (BFPC), there is interest in exploring expanded paraprofessional staffing models to support nutrition services with low risk WIC clients (27–29).

Through a landscape policy analysis of 23 WIC state agencies, we explored the potential to engage paraprofessionals in nutrition services provided by WIC. Consistent with the decentralized nature of WIC administration (31), substantial variations were observed across jurisdictions reviewed. For example, a total of 242 unique job titles were identified, with as few as three job titles in one state, and as many as 19 in another. Notably, although more than half of all job titles were related to the provision of nutrition services, the term “paraprofessional” appeared infrequently and was not used consistently across states. Only seven of the 23 PPMs (30.4%) referenced paraprofessionals in non-breastfeeding roles, and only one standardized title (CPPA) explicitly incorporated the term in any jurisdiction. Further, we found substantial variations in who performs nutrition-related functions, what qualifications are requires, which functions may be delegated and whether competency-based pathways are available. These findings suggest that paraprofessional contributions to nutrition services may be under-recognized within existing workforce structures, and that there may be strategic opportunities to strengthen the WIC workforce through broader adoption of a paraprofessional model.

Despite limited use of the term “paraprofessional,” our findings indicate that many nutrition-related positions may be open to paraprofessionals in practice. Across all nutrition-service titles reviewed, more than one-third (37.4%) appeared to be potentially accessible to individuals without a college degree in at least some jurisdictions. This suggests that, although the term “paraprofessional” is not used in a ubiquitous manner, there may be opportunities for individuals without formal nutrition or health-related credentials or a college degree to contribute meaningfully to WIC nutrition-service delivery. Additionally, beyond task delegation, there are also opportunities for organizational design. The effectiveness of paraprofessional workforce models may depend not only on individual competencies but also on role definition, supervision, referral pathways, team composition, accountability, and organizational processes.

These findings are poignant considering WIC’s experience adopting a paraprofessional model for breastfeeding-service delivery. In 2004, following strong pilot evidence, WIC implemented the BFPC program, which employs paraprofessionals, often current or former WIC participants, to provide breastfeeding support to pregnant and postpartum women (42–44). Evaluations demonstrate improvements in staffing capacity, participant satisfaction, continuity of care, and cost-effectiveness (45–48). These successes illustrate the potential value of a structured or standardized approach to better engage paraprofessionals in expanded WIC services (31, 49, 50); our work shows that while some WIC state agencies appear to have already integrated paraprofessionals into their model of delivery for nutrition services, there are opportunities to broaden this approach. For example, we identified several nutrition-related roles across multiple jurisdictions that appear to be open to staff without a college degree. Across these roles, we identified 32 key functions that could be fulfilled more systematically by paraprofessionals, including screening for eligibility, conducting certification, collecting anthropometric and hematological measurements, assessing nutrition risk, providing nutrition education, issuing food benefits, documenting participant encounters, and making referrals. These observations also suggest value in implantation projects to evaluate whether specific tasks can be safely and effectively allocated according to demonstrated competencies, with appropriate supervision and accountability.

Several priorities for future research and policy development emerge from this work. First, there is a need to better understand barriers and facilitators to adoption of nutrition paraprofessional roles within WIC. Although federal guidance regarding paraprofessional nutrition assistants was issued by FNA in 1991 (30), little is known about how these recommendations were implemented or what factors influenced adoption across states (31, 49, 50). Further research involving federal, state, and local stakeholders could help clarify opportunities and challenges associated with broader integration of paraprofessionals into nutrition service delivery.

Second, innovative WIC workforce models that incorporate “nutrition paraprofessionals” should be documented and evaluated. Our work showed considerable variation in staffing structures across state agencies, including some that appear to engage “nutrition paraprofessionals.” Consistent with evidence from other sectors (36, 39), evaluating local innovations may identify promising practices that could be adapted and scaled across WIC programs. Qualitative and implementation-focused research could be particularly valuable in understanding the effects of these models on recruitment, retention, workforce satisfaction, and participant outcomes.

Third, greater consistency in job titles, role definitions, and career pathways may strengthen workforce development. For example, although federal policy establishes requirements for CPAs (24), substantial variations appear to exist in how CPA-related functions are operationalized across jurisdictions. In some state agencies, few positions fill this role, and all require university credentials; in others, there appear to be multiple job titles that fill CPA-related functions, and opportunities for training and advancement for staff without a college degree. Greater consistency in competencies, role definitions, and career pathways may facilitate workforce development. In doing so, FNA might leverage learnings from the BFPC program (43, 44), and define competencies, training requirements, scopes of practice, and role expectations for nutrition paraprofessionals. The positions identified in this study, including Clerk, CPPA, Nutrition Assistant, and Nutrition Educator, may provide a useful starting point for further exploration.

Finally, competency-based training models warrant further attention. Six state agencies reviewed presented training and mentoring pathways to prepare paraprofessional staff to perform nutrition-related functions. These findings align with broader evidence supporting competency-based approaches to workforce development and task-sharing (38–41). Evaluation of existing models could determine whether they offer an effective strategy for expanding workforce capacity while maintaining service quality, particularly in areas experiencing persistent recruitment challenges, including rural communities (25, 26).

## Limitations

While this study provides insight into a focal area for workforce development, there are limitations. First, the analysis included only 23 of the 88 WIC state agencies in the U.S. While these states were sampled systematically to ensure representation by geography, size, and economics, the findings do not represent all workforce models currently in use. Second, findings were based on publicly available PPMs and related web-based materials, which may not fully reflect workforce practices at the local agency level. Third, the level of detail provided in policy documents varied across jurisdictions which may have limited the comparability of job functions, qualifications, and training pathways in some cases. Fourth, degree requirements that were ‘Not Listed’ were interpreted as absence of college degree. Finally, this study was descriptive in nature and did not evaluate the implementation or effectiveness of different staffing models, competency-based training approaches, or paraprofessional integration strategies.

Despite these limitations, this study has several strengths. Jurisdictions were selected using a stratified sampling approach to capture geographic and administrative diversity across the WIC system. Further, the review applied a systematic methodology to classify 242 unique job titles into standardized workforce categories and to identify common nutrition-service functions across jurisdictions. To our knowledge, this is the first cross-jurisdictional analysis to examine the potential role of paraprofessionals within WIC nutrition services, providing a foundation for future workforce research, policy development, and program innovation.

## Conclusion

There are opportunities to engage paraprofessionals in the delivery of nutrition-service roles to help strengthen WIC workforce capacity. Our review identified a variety of job titles, qualification requirements, delegated responsibilities, and competency-based training pathways that allow staff to assume progressively greater nutrition-service responsibilities. The findings suggest that expanding workforce capacity may depend not simply on increasing the number of staff, but on designing clear role definitions, competency-based training pathways, supervision structures, and organizational models that appropriately allocate nutrition-service functions across workforce categories. Further research is needed to identify promising practices, evaluate workforce outcomes, and inform workforce-development strategies that improve access to nutrition services while maintaining quality of care.

## Data Availability

The original contributions presented in the study are included in the article/Supplementary material, further inquiries can be directed to the corresponding author.
